# Solid Dispersions Obtained by Ball Milling as Delivery Platform of Etodolac, a Model Poorly Soluble Drug

**DOI:** 10.3390/ma17163923

**Published:** 2024-08-07

**Authors:** Anna Czajkowska-Kośnik, Iwona Misztalewska-Turkowicz, Agnieszka Zofia Wilczewska, Anna Basa, Katarzyna Winnicka

**Affiliations:** 1 Department of Pharmaceutical Technology, Medical University of Białystok, Mickiewicza 2c, 15-222 Białystok, Poland; kwin@umb.edu.pl; 2 Department of Organic Chemistry, University of Białystok, Ciołkowskiego 1K, 15-245 Białystok, Poland; i.misztalewska@uwb.edu.pl (I.M.-T.); agawilczuwb@gmail.com (A.Z.W.); abasabo@gmail.com (A.B.)

**Keywords:** etodolac, solid dispersion, ball milling, polyvinylpyrrolidone, hypromellose, copovidone, Poloxamer, solubility improvement

## Abstract

Poor water solubility of drugs is a limiting factor for their bioavailability and pharmacological activity. Many approaches are known to improve drug solubility, and among them, the physical method, solid dispersions (SDs), is applied. SDs are physical mixtures of a drug and a carrier, sometimes with the addition of a surfactant, which can be obtained by milling, cryomilling, spray-drying, or lyophilization processes. In this study, solid dispersions with etodolac (ETD-SDs) were prepared by the milling method using different carriers, such as hypromellose, polyvinylpyrrolidone, copovidone, urea, and mannitol. Solubility studies, dissolution tests, morphological assessment, thermal analysis, and FTIR imaging were applied to evaluate the SD properties. It was shown that the ball-milling process can be applied to obtain SDs with ETD. All designed ETD-SDs were characterized by higher water solubility and a faster dissolution rate compared to unprocessed ETD. SDs with amorphous carriers (HPMC, PVP, and PVP/VA) provided greater ETD solubility than dispersions with crystalline features (urea and mannitol). FTIR spectra confirmed the compatibility of ETD with tested carriers.

## 1. Introduction

Drug solubility is one of the limiting factors to obtain the preferred dosage form. All substances, before they cause the expected therapeutic effect, must be dissolved in the surrounding medium to penetrate the biological membranes of the body [1,2,3,4,5]. Unfortunately, most drugs have limited solubility in water, and many approaches have been developed to improve solubility, among them various types of physical (micronization and nanosuspensions) and chemical (addition of surfactants, pH changes, and complexation) modifications. One of the ways to increase drug solubility is using solid dispersions (SDs) [6,7]. SDs are the solid-state systems of one or more drug substances in an inert carrier or matrix. In such two-component structure, it is necessary to ensure adequate dispersion of the drug and its stability. By using SDs, both the solubility and bioavailability of the drug substance can be improved. SDs are a promising option to increase the solubility of drugs belonging to class II of the BCS (Biopharmaceutical Classification System), which includes drugs with limited solubility in water but characterized by good permeation through biological membranes [8,9]. It is presumed that the use of SDs permits an increase in the dissolution area and thus an improvement of bioavailability. In addition, by reducing the particle size of the drug and increasing the wetting and porosity of SDs, the dissolution rate is intensified. SDs could be manufactured by various techniques, such as milling, solvent evaporation, melting, and melting–solvent evaporation methods. Among solvent evaporation methods, a lot of modifications are known, e.g., spray-drying, lyophilization, supercritical fluid, co-precipitation, and electrospinning techniques. Hot-melt extrusion and melt agglomeration are examples of melting methods [7,10,11,12,13]. Ball milling is a simple method for SD preparation, in which the drugs and carriers are mixed for a long time without solvent and heating. During milling, the reduction in particle size and transformation of the drug from a crystalline to an amorphous form can be achieved [14,15].

**Figure 1:**
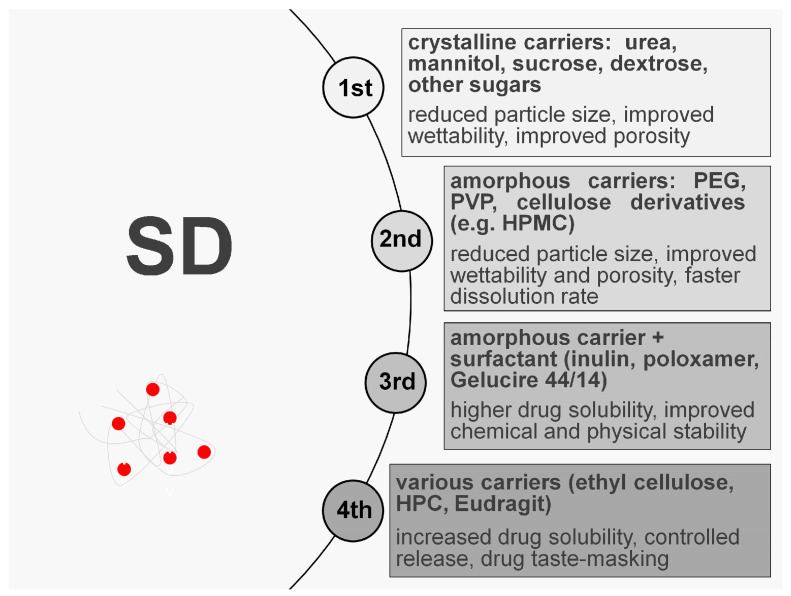
The generations of SDs [11,22].

Based on the type of carrier used, four classes of SDs can be identified. In the first generation of SDs, the drug and carrier are in crystalline form. The most commonly used carriers for this generation are urea and sugars, such as dextrose, sucrose, galactose, sorbitol, maltose, xylitol, mannitol, or lactose. The main purpose of mixing the drug substance with the above carriers is to improve solubility, which is achieved primarily by reducing the particle size of the drug, and by better wettability. However, the first generation of SDs presents difficulties, such as drug recrystallization, phase separation, and physical instability, over time [14,16]. In the second generation of SDs, amorphous carriers (especially polymers) are used, e.g., polyethylene glycols (PEG), povidone, polyvinylpyrrolidone (PVP), cyclodextrins (CD), or cellulose derivatives—hydroxypropylmethylcellulose (HPMC) [17,18]. The third generation of SDs is based on the polymer as a carrier in combination with a surfactant. As surfactants, polyoxyethylene and polyoxypropylene copolymers (Poloxamer), glycerol behenate (Compritol 888), or polyethylene glycol glycerides composed of mono-, di-, and triglycerides and polyethylene glycol mono- and di-esters (Gelucire 44/14) are used. The third generation of SDs, due to the addition of surfactants, enhances the solubility to a higher degree than the second generation, where only polymers are applied. The use of a surfactant compound also improves the stability of drug formulations [19,20]. The fourth generation of SDs are controlled-release dispersions. The carriers are ethyl cellulose, polymethacrylate (Eudragit), or hydroxypropyl cellulose. The fourth generation of SDs is used for drugs that are poorly water-soluble and have a short biological half-life and provides the increase in their solubility and achieves controlled release [10,21]. The described generations of SDs are illustrated in Figure 1.

PVP (polyvinylpyrrolidone) is one of the most widely used polymers in the preparation of SDs. It is soluble in water, ethanol, chloroform, and isopropyl alcohol. PVP is available in different grades and is characterized by varied molecular weight and viscosity values. The dissolution rate of SDs with PVP depends on the molecular weight of the type used—as the molecular weight increases, the dissolution rate decreases, due to the increase in viscosity and PVP swelling. In acidic pH, PVP shows a structural transformation, i.e., at pH 1.2, PVP (form I) converts to the gem-diol (form III), where the –OH group can participate in the formation of hydrogen bonds with the drug, enhancing its aqueous solubility [23]. In this study, Kollidon 25 (brand name of PVP), characterized by a lower molecular weight (28,000–34,000 g/mol) and low viscosity (10 mPaꞏs at 25 °C), was used. PVP belongs to amorphous carriers, so it forms SDs of the second generation [24,25,26]. Pharmacoat 606 (hydroxypropylmethylcellulose, HPMC) is a cellulose derivative, soluble in water and in organic solvents, including ethanol, methanol, propanol, and dichloromethane. SDs with HPMC can be prepared by lyophilization, spray drying, and solvent evaporation. HPMC is a highly hygroscopic polymer, and it strongly inhibits drug recrystallization. HPMC belongs to the amorphous carriers and, similar to PVP, forms SDs of the second generation [25,27]. Urea is an organic nitrogen compound, well soluble in water and organic solvents. It is thermally unstable. SDs with urea have higher solubility and dissolution rates than those prepared with mannitol. Urea is a crystalline carrier, and SDs prepared with it belong to the first generation. Mannitol is a water-soluble sugar with extremely high thermal stability and low molecular weight. It is compatible with most drugs and can be used to produce SDs by thermal methods and methods with organic solvents. It belongs to the crystalline carriers that form the first generation of SDs [27]. PVP/VA (Kollidon VA64, copovidone) is a copolymer of polyvinylpyrrolidone and polyvinyl acetate in a ratio of 6:4. It is an amorphous, water-soluble polymer and it forms SDs of the second generation. Due to the large difference between its glass transition and degradation temperatures, it can be used in the preparation of SDs of drugs with both low and high melting points. PVP/VA strongly inhibits drug recrystallization, and is less hygroscopic than PVP [28,29].

Etodolac (ETD) represents a non-steroidal anti-inflammatory drug (NSAID). Chemically, it is an acetic acid derivative, similar to diclofenac or indomethacin. Similar to all drugs in this group, it is characterized by analgesic, anti-inflammatory, and antipyretic effects and is selective toward cyclooxygenase 2. ETD is a weak acid (pKa 4.65); therefore, at higher pH values (pH > pKa), the ionized form of the drug dominates (the pyrano group of ETD is ionized), which improves ETD’s aqueous solubility. It is characterized by a molecular weight of 287.35 g/mol, and belongs to class II of BCS, which means it has poor water solubility (0.016 mg/mL), but high permeability across lipophilic barriers (log P 2.5). Its half-life is relatively short, about 7 h, making it necessary to take ETD frequently for oral therapy in order to keep it effective, which can lead to side effects [30,31,32,33]. Due to its therapeutic properties, it is used in the treatment of pain, rheumatoid arthritis, and osteoarthritis. ETD is commercially available in the form of oral preparations (tablets, capsules, and extended-release tablets) [34,35].

The purpose of this study was to prepare the solid dispersions with ETD (ETD-SDs) and to estimate their properties. SDs were obtained by the ball-milling technique, using different carriers. Some of the designed SDs contained a surfactant compound—they represented the third generation of SDs. During the evaluation of SDs, the drug content, solubility in water and in buffer pH 7.4, the dissolution profile, morphological analysis, DSC, and FTIR studies were performed.

## 2. Materials and Methods

### 2.1. Materials

Materials were obtained from commercial sources. ETD was supplied from Xi’an Health Biochemical Technology Co. (Xi’an, China). Kollidon 25 (polyvinylpyrrolidone, PVP) and Kollidon VA 64 (polyvinylpyrrolidone-polyvinyl acetate copolymers, PVP/VA) were provided by BASF (Burgbernheim, Germany). Pharmacoat 606 (hypromellose) was obtained from Shin-Etsu Chemical Co. (Niigata, Japan) and Poloxamer 407 (poly(ethylene glycol)-block-poly(propylene glycol)-block-poly(ethylene glycol)) from Sigma Aldrich (Steinheim, Germany). Urea, mannitol, and potassium dihydrogen phosphate were provided by Chempur (Piekary Sląskie, Poland), and disodium hydrogen phosphate by P.P.H. Stanlab (Lublin, Poland). Water for HPLC analysis was prepared by a Milli-Q Reagent Water System (Millipore, Billerica, MA, USA), and purified water by the reverse osmosis and deionization method (apparatus type WCR-RO10-DPS, Cobrabid-Aqua, Warszawa, Poland). Acetonitrile (J.T. Baker, Deventer, The Netherlands) was of HPLC grade, and other chemicals were of analytical grade.

### 2.2. High-Performance Liquid Chromatography Analysis

Analysis of ETD content was conducted by high-performance liquid chromatography (HPLC), with modification [36]. Agilent Technologies 1260 Infinity equipment (Agilent, Waldbronn, Germany) and the Eclipse XDB C18 column (5 µm, 4.6 × 250 mm; Agilent Technologies, Böblingen, Germany) were used for measurement of ETD levels. The method conditions were as follows: the mobile phase was acetonitrile and 0.02 M phosphate buffer with pH 6.0 (50:50, *v*/*v*) in an isocratic flow (1 mL/min), with temperature maintained at 25 °C. The analytical wavelength of 225 nm was based on the UV-Vis spectrum of ETD in the wavelength range from 150 to 400 nm. The retention time of ETD was 3.4 min.

A standard stock solution of ETD was obtained by dissolving 10 mg of the substance in 10 mL of acetonitrile (1000 µg/mL). Then, sufficient amounts of the standard stock solution were taken in a volumetric flask and then diluted with the mobile phase to a concentration in the range of 0.5 to 7.5 µg/mL. Every sample was prepared in triplicate.

The linearity of the method was estimated by linear least squares regression analysis of a plot of peak area as a function of ETD concentration. The standard calibration curve was linear in the range from 0.5 to 7.5 µg/mL (R^2^ = 0.999), with precise within-day and between-day requirements, as represented by the values of the relative standard deviation (ranging from 0.34 to 2.93% for a concentration of 5 µg/mL). The limit of quantification (LOQ) was 0.47 μg/mL and limit of detection (LOD) was 0.15 μg/mL. The robustness of the HPLC method was analyzed by slight, targeted changes in the method, as variations in mobile phase pH, temperature, and percentage of acetonitrile. There were no obvious changes (less than 5%) in ETD chromatograms, so the used HPLC method was highly reliable.

### 2.3. Preliminary Studies—Development of the Preparation Method, Formulation, and Characterization of the 1st and 2nd Generation of ETD-SDs

#### 2.3.1. Preparation of ETD-SDs

**Figure 2:**
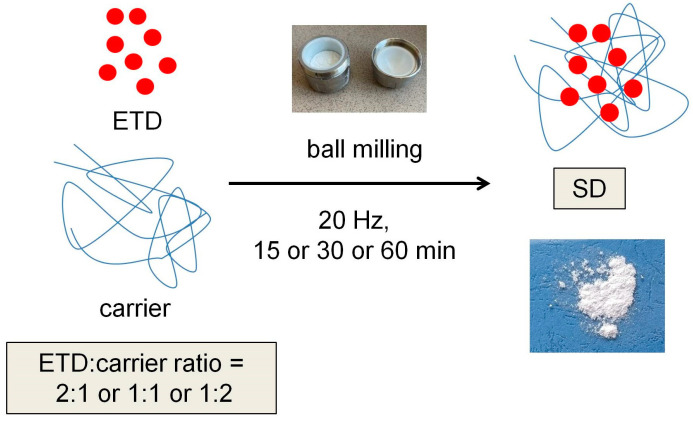
Preparation of ETD-SDs by the ball-milling method.

ETD-SDs were obtained by milling using the Retsch GmbH MM 400 ball mill (Haan, Germany). Mixtures of ETD and carriers in variable ratios (2:1, 1:1, or 1:2) were initially mixed in a Multi-Rotator (100 rpm, 10 min). The prepared formulations were then transferred to the steel jars containing 3 zirconia spheres (10 mm in diameter). The milling process was carried out for a defined time (15, 30, or 60 min), in 15 min cycles, operated at a frequency of 20 Hz (Figure 2). The milling frequency value of 20 Hz was selected in the preliminary tests.

The prepared SDs were stored in tightly closed containers until analysis. Formulations F1, F2, and F3 contained pure ETD, with no carrier added, while formulations F4–F48 comprised urea, mannitol, or different polymers, such as PVP (polyvinylpyrrolidone), Pharmacoat 606 (hydroxypropylmethylcellulose), and PVP/VA (polyvinylpyrrolidone-polyvinyl acetate copolymer). SD composition and time of milling are presented in Table 1.

#### 2.3.2. Solubility Studies

The solubility tests were performed by mixing the purified water with excess amounts of SD formulations. The flasks were shaken for 1 min using the Vortex Genie-2 shaker (Scientific Industries, Bohemia, NY, USA). Then, the formulations were placed in a water bath at 25 ± 1 °C and shaken at 250 rpm for 24 h. After the indicated time, the samples were centrifuged at 4000 rpm for 10 min, and then filtered through cellulose acetate filters (0.2 µm). In the next step, the filtrates were diluted with mobile phase and the content of ETD in each sample was determined using the HPLC method, as described in Section 2.2. Three replicates were performed for each formulation, and the results obtained are presented as averages with deviations.

#### 2.3.3. Differential Scanning Calorimetry (DSC)

Differential scanning calorimetry (DSC) was performed using a DSC TEQ2000 unit (TA Instruments, New Castle, DE, USA). Samples of 3–5 mg were placed in aluminum crucibles, followed by heating at a temperature from 40 °C to 210 °C at a scan rate of 10 °C/min and nitrogen flow rate of 20 mL/min. In the study, pure ETD, pure carriers, and their physical mixtures were used as references.

### 2.4. Solid Dispersion with ETD—2nd and 3rd Generation

#### 2.4.1. Preparation of ETD-SDs

After preliminary tests (point 2.3) based on the formulations prepared at different ETD:carrier ratios and the milling time, the optimal SD formulations were selected. In the next step, 10 formulations were obtained by milling using the Retsch ball mill. Formulations S1–S6 contained ETD, a carrier, and a surfactant, Poloxamer 407 (3rd generation), formulations S7–S9 contained only ETD and a carrier (2nd generation), while formulation S10 was a mixture of ETD and Poloxamer. The milling process was carried out in the ball mill for 60 min (intervals of every 15 min were introduced to avoid overheating of the mixture) at a frequency of 20 Hz. The prepared SDs with ETD were stored in tightly sealed dark-glass vessels. The composition of the prepared ETD-SDs is shown in Table 2.

#### 2.4.2. Drug Content Studies

Samples of 10 mg of ETD-SDs were weighed into a volumetric flask and filled with acetonitrile (10 mL). The prepared samples were transferred to a water bath and shaken (250 rpm) for 24 h at room temperature (20 ± 1 °C). After this time, SD formulations were subjected to filtration (0.22 µm) and then, after dilution, analyzed by HPLC (Section 2.2).

#### 2.4.3. Solubility Analysis and Dissolution Testing

The solubility of ETD in water and phosphate buffer pH 7.4 was performed according to the method described in Section 2.3.2. The dissolution assay was conducted using the USP Dissolution Apparatus II (Erweka DT 600D, Heusenstamm, Germany). SD formulations (containing 50 mg of ETD) were introduced into beakers containing 100 mL of acceptor fluid–phosphate buffer pH 7.4. Phosphate buffer pH 7.4, the same as buffers with lower pH (e.g., 6.8 or 5.5), is used as acceptor medium in drug dissolution studies designed for topical administration [37,38,39]. Phosphate buffer pH 7.4 was also chosen due to the higher ETD solubility (1.51 mg/mL) than what was observed in acetate buffer pH 5.5 (0.37 mg/mL). The assay was conducted at 37 ± 1 °C, with a stirring speed of 100 rpm. Samples of acceptor fluid were taken after 5, 10, 15, 20, 30, 45, and 60 min of the test and pure buffer was added after each sampling. The dissolution rate test was conducted in triplicate (mean ± SD). In order to compare the dissolution profiles of ETD-SDs with unprocessed drug, a mathematical analysis was carried out, to determine the dissolution efficiency (DE, %), mean dissolution time (MDT, min), and the similarity factor f_2_, using the DD-Solver program (add-in program for Microsoft Excel) [40]. The kinetic interpretation was also performed to verify the drug dissolution model. The analysis was conducted with zero-order, first-order, and Higuchi models. The assessment of the best model was based on the value of the correlation coefficient (R^2^), the highest value of which indicated the preferable model [41].

#### 2.4.4. Morphological Assessment

The morphology, shape, porosity, and size of SDs with ETD were evaluated using a scanning electron microscope (SEM; Hitachi S4200, Tokyo, Japan). Before imaging, each sample was coated with a gold layer. The ETD-SDs were observed under magnifications of 2000×.

The morphology of SDs was also investigated by transmission electron microscopy (TEM; Tecnai G2, FEI Company, Hillsboro, OR, USA) operating at 200 kV. In order to perform the TEM observations, 2% phosphotungstic acid (PTA) was added to SDs. The sample was shaken for 30 s and was applied to the copper holey carbon-coated grid (300 mesh). After drying, the samples were observed at different magnifications.

#### 2.4.5. Thermal Analysis

The DSC study of formulations S1–S10 was conducted according to Section 2.3.3. TGA measurements were performed using the Mettler Toledo Star TGA/DSC unit (Leicester, UK). The samples, weighing 2–3 mg, were placed in an aluminum oxide crucible and heated from 50 °C to 600 °C. The heating rate of 10 °C/min and an argon flow rate of 40 mL/min were applied. As empty pan was used as a reference. TGA was carried out for pure ETD, urea, the physical mixture of ETD and urea (1:1), and SDs with ETD and urea (1:1) to evaluate the interaction between the drug and carrier or to assess the degradation process of the compounds.

#### 2.4.6. Attenuated Total Reflectance Fourier Transform Infrared Spectroscopy (ATR-FTIR)

FTIR analysis was carried out using the Thermo Scientific Nicolet 6700 FTIR spectrophotometer (Waltham, MA, USA) equipped with diamond attenuated total reflectance. Spectra were rationed against the background spectrum, and 32 scans from 400 to 4000 cm^−1^ were conducted.

### 2.5. Statistical Analysis

The results are presented as mean values with standard deviations (mean ± SD), calculated using Microsoft Excel. Statistical analysis of the results was carried out by Statistica 13.3 software (StatSoft, Krakow, Poland). The normality of the data was tested using the Shapiro–Wilks test. Data on solubility in water and buffer were characterized by normal distributions, and the Tukey test was used to examine significant differences. The results for the dissolution rate were not normally distributed, so the Kruskal–Wallis test was used to assess significant differences. Differences were defined as significant when *p* was < 0.05. Three-dimensional (3D) response surfaces were obtained from the Statistica 13.3 program to determine optimal points—the effect of carrier concentration and Poloxamer on SD properties.

## 3. Results and Discussion

### 3.1. Preliminary Studies—1st and 2nd Generation of ETD-SDs

Forty-eight formulations were prepared with ETD as a model drug, with different carriers (PVP, Pharmacoat 606, urea, mannitol, and PVP/VA), ETD:carrier ratios (2:1, 1:1, and 1:2), milling times (15, 30, and 60 min), and with a constant milling frequency of 20 Hz. Formulations F1–F3 contained only ETD, while the others included carriers: F4–F12—PVP, F13–F21—Pharmacoat, F22–F30—urea, F31–F39—mannitol, and F40–F48—PVP/VA.

#### 3.1.1. Solubility

**Figure 3:**
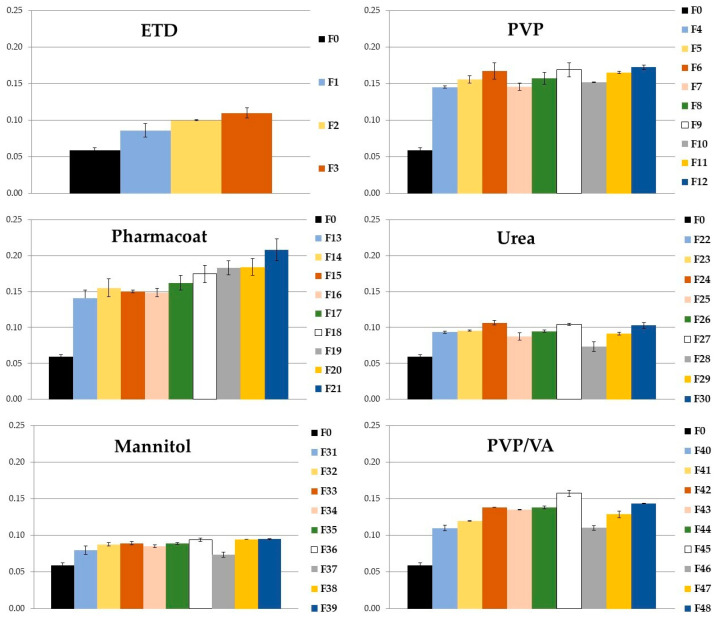
ETD water solubility in SDs: F4–F12—SDs with PVP, F13–F21—SDs with Pharmacoat, F22–F30—SDs with urea, F31–F39—SDs with mannitol, and F40–F48—SDs with PVP/VA, and solubility of control: F0—unprocessed ETD (the first column of each chart), and F1–F3—milled ETD for 15, 30, and 60 min. The *y*-axis presents the solubility of ETD, expressed as mg per mL.

The prepared ETD-SDs were tested for drug solubility, and unprocessed ETD was used as the reference (Figure 3). SD formulations with ETD contained various carriers: PVP, Pharmacoat, urea, mannitol, and PVP/VA. Each carrier was examined at various ratios to ETD (2:1, 1:1, and 1:2) and different milling times (15, 30, and 60 min). The solubility enhancement was observed for each formulation that had been milled (F1–F48) compared to unprocessed ETD (F0; *p* < 0.05). There was a noticeable difference in the solubility of ETD in SDs depending on the type of the carrier—higher solubility of ETD was observed in SDs with amorphous carriers, i.e., PVP, Pharmacoat 606, and PVP/VA. The use of amorphous polymers increased the solubility of ETD by 2–3 times (the highest values were achieved for Pharmacoat in the range from 0.141 to 0.208 mg/mL). The use of crystalline carriers—urea and mannitol—increased ETD solubility only to 0.107 mg/mL (the highest value was found for ETD:urea in a ratio of 1:1). Based on the results, it can be concluded that the preparation of SDs with ETD improved the solubility in aqueous solution from 0.006% to 0.02% (Pharmacoat as a carrier). Studies conducted by Karolewicz et al. [42] and other research confirm the results obtained in the present work and indicate that SDs with amorphous carriers provide the higher drug solubility [43,44,45].

The effect of milling time (from 15 min to 60 min) on ETD solubility was observed. For pure ETD formulations (F1–F3), without carriers, the highest solubility was shown in formulation F3 (0.11 mg/mL), which was milled for 60 min. A similar trend was observed for the other formulations, where ETD was blended with the carriers. The largest increase in ETD solubility after 60 min of milling was observed for PVP/VA (an increase of 0.4–0.5 mg), so it can be concluded that the time of the milling process had an impact on the quality of the obtained SDs: the longer the time of milling, the higher the ETD solubility observed. At the same time, for most SDs, no effect of the concentration of the carrier used (from 33.3% to 66.6%) on drug solubility was noted. However, for the formulation with Pharmacoat, the increase in carrier concentration to 66.6% resulted in the increase of ETD solubility, from 0.15 mg/mL to 0.21 mg/mL (milling for 60 min). Thus, it can be concluded that the best ETD solubility was provided by SDs formed with amorphous carriers, especially with PVP or Pharmacoat 606.

#### 3.1.2. Thermal Evaluation

Differential scanning calorimetry (DSC) is an analytical technique used for identifying the changes in the samples under examination as a function of time and temperature. During the temperature change, DSC measures the amount of excessive heat radiated or absorbed by the sample, based on the temperature difference between the tested and reference materials. This method is used to estimate the drug melting point, drug stability, the purity, to detect polymorphic forms of the drug, and to detect the incompatibilities between drugs and carriers, as well as to determine the form of the active substance—crystalline or amorphous. Analysis of the thermograms (DSC curves), i.e., shift, appearance, or disappearance of exothermic and/or endothermic peaks, enables assessment of the transformations taking place in the tested material [46,47,48].

The thermograms were obtained for pure ETD, pure carriers (PVP, Pharmacoat, urea, mannitol, and PVP/VA), physical mixtures of ETD and carriers, and for SDs (F4–F48). The DSC curve of unprocessed ETD showed a sharp endothermic peak at 153 °C, corresponding to the melting point of the drug, and indicated its crystalline state. The carriers, such as PVP, Pharmacoat 606, and PVP/VA, showed straight lines on the DSC thermograms (no peaks), which indicated the amorphous properties of these substances, as confirmed by the literature data [49]. In contrast, DSC curves of carriers, such as urea and mannitol, were characterized by endothermic peaks at 136 °C and 168 °C, respectively, which corresponded to their melting points. In the case of urea, a line breakdown above 160 °C was observed. This relationship was also noted during the examination of SD formulations with urea, which may indicate changes related to its degradation. Literature data reported that urea degrades at temperatures above 175 °C [50,51].

The analysis of DSC curves of pure ETD (formulations F1–F3, milling for 15, 30, and 60 min, respectively) showed endothermic sharp peaks in the temperature range of 150–153 °C (Figure S1). The milling process of pure ETD, in spite of the time of milling, did not lead to changes in the form of the compound (amorphization) or a significant change in its melting point.

**Figure 4:**
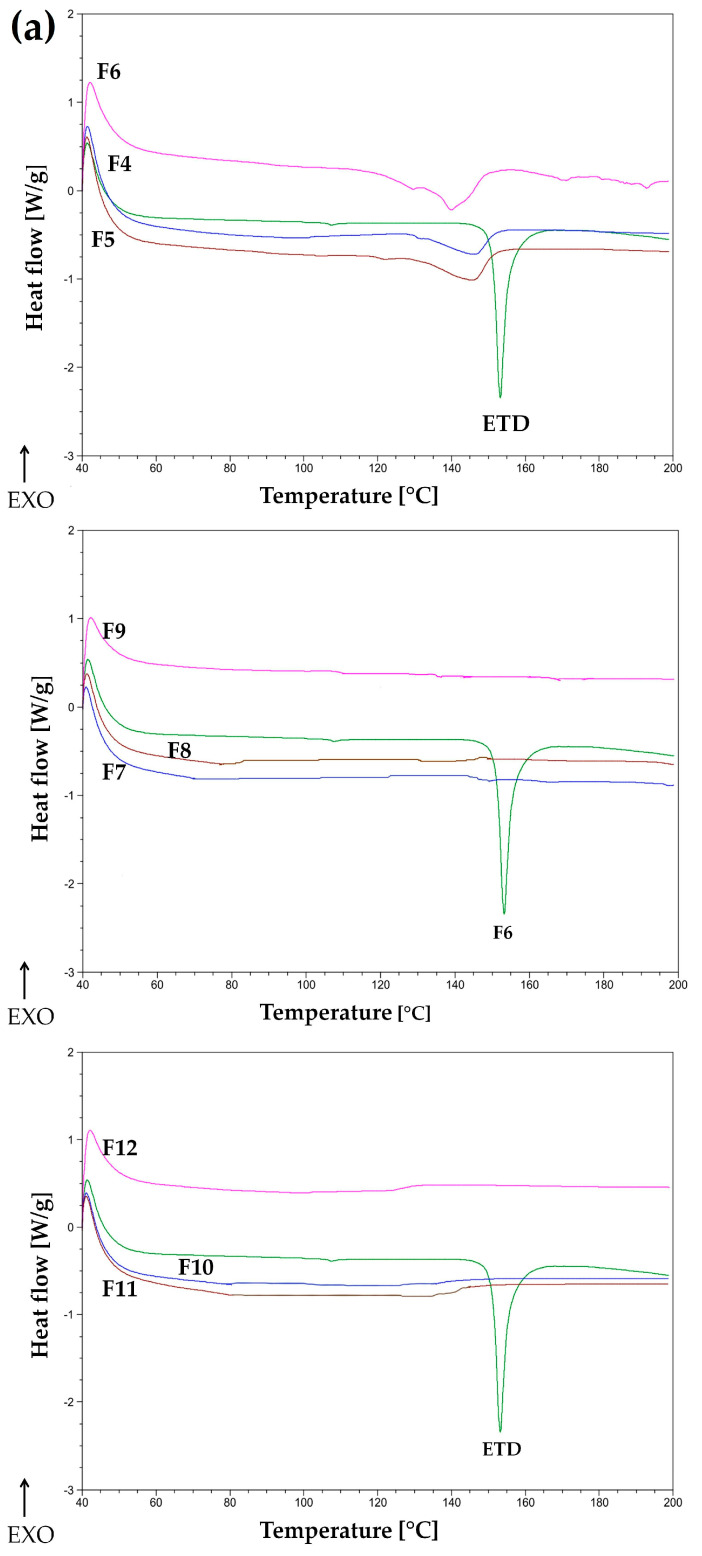

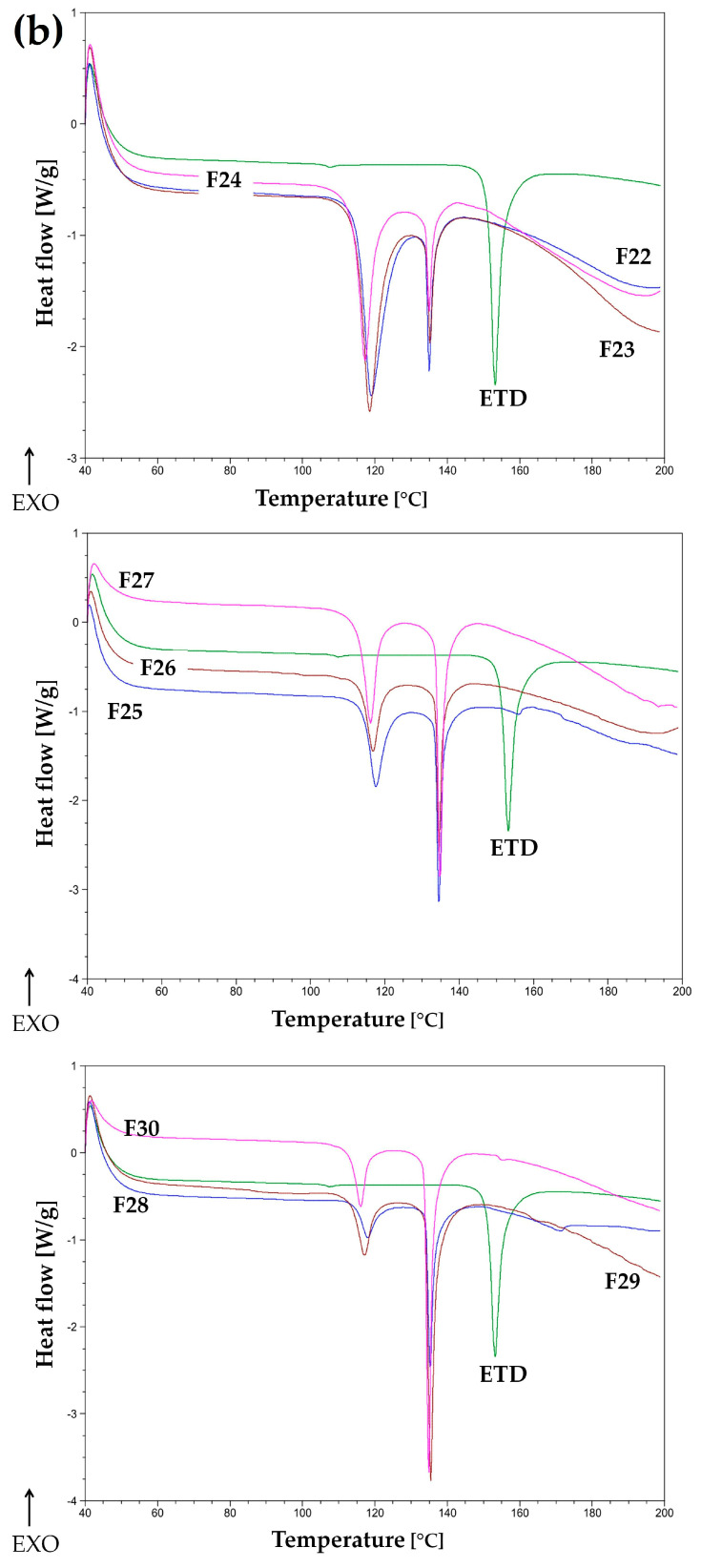

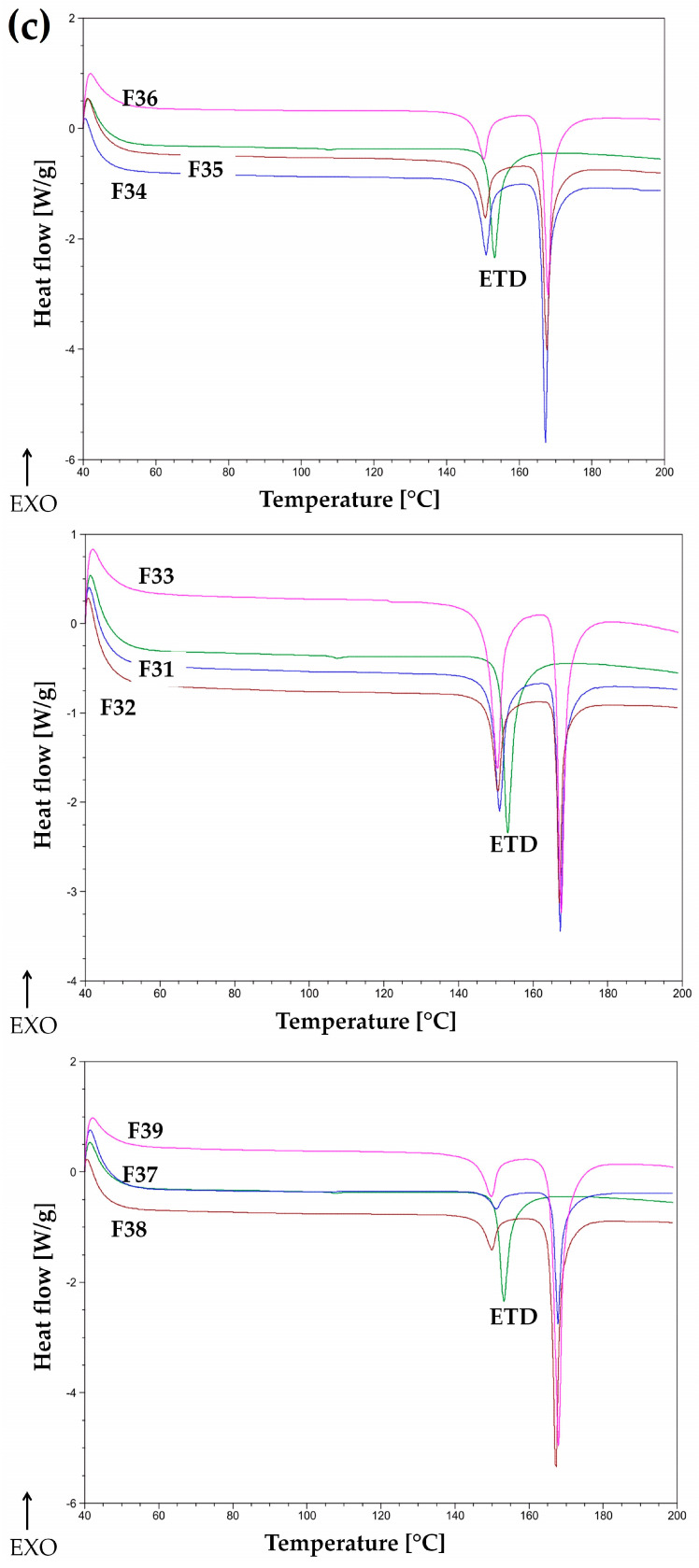
DSC curves of ETD-SDs with carriers: (**a**) PVP, (**b**) urea, and (**c**) mannitol. The ETD:carrier ratios were 2:1 (F4–F6; F22–F24; F31–F33), 1:1 (F7–F9; F25–F27; F34–F36), and 1:2 (F10–F12; F28–F30; F37–F39).

SD formulations prepared with PVP (F4–F12, Figure 4a) showed a gradual loss of the sharp endothermic peak of ETD, which was invisible in formulation F7 (ratio of drug:carrier was 1:1, and a milling time of 15 min). The formulations F4–F6 did not show a sharp peak for ETD, which existed in the physical mixture or in pure ETD. Only a small endothermic peak with a shift in the melting point of ETD below 150 °C was visible. This confirmed the influence of the polymer concentration on the quality of SD—the use of a carrier:drug ratio of 1:1 or 2:1 might cause the ETD to transition to an amorphous state. The reduction in the sharp peaks and the decrease in the melting point can be attributed to the formation of the amorphous state of ETD in SDs and the change in its crystalline form and/or to the dissolution of the ETD in the molten carrier [52]. A similar relationship to SD with PVP was noted for Pharmacoat (formulations F13–F21, Figure S2). At a carrier concentration of 33.3%, a slight shift in the ETD melting point was observed (about 150 °C), while a greater peak reduction (loss of ETD peak sharpness) was observed at a Pharmacoat concentration of 50%. Increasing the amount of polymer to 66% resulted in a complete disappearance of the peak characteristic for ETD [53,54].

All DSC curves of SD with urea (formulations F22–F30, Figure 4b) demonstrated the sharp endothermic peak typical for urea—at 136 °C. Significantly, the endothermic peak characteristic for ETD (at 153 °C) did not exist, while a new peak appeared at around 120 °C. The relationship observed was that the peak at 120 °C decreased as the amount of carrier increased (from 33% to 66%). The occurrence of a new peak at 120 °C and the loss of the peak for ETD at 153 °C might indicate interactions between ETD and urea. Moreover, thermograms for SD with urea showed a line collapse above 160 °C (probably degradation of the compound) [51].

Analyzing the DSC curves of mannitol–SD formulations (formulations F31–F39, Figure 4c), a sharp endothermic peak at 168 °C—characteristic of mannitol—and a peak for ETD were detected. The results indicated that ETD in SDs with mannitol remained in crystalline form [14,55]. The formulations with PVP/VA (formulations F40–F48, Figure S3) exhibited a lack of an endothermic peak for ETD for all SD, probably as a consequence of ETD’s transformation into an amorphous form [56,57].

#### 3.1.3. Statistical Analysis

Statistical analysis of the results (central composite design—response surface) was performed, and an ANOVA test was set up with a statistical significance level of 95% (α = 0.05). The effect of input factors (carrier type, carrier concentration, and milling time) on ETD solubility was determined. Based on this analysis, it was found that both milling time (*p* = 0.03) and carrier type (*p* = 0.00002) had statistically significant effects. In contrast, the concentration of the carrier had no statistically significant effect on ETD’s solubility (*p* = 0.29). Among all tested carriers, Pharmacoat and PVP provided the best solubility for ETD, whereas an increase in the milling time from 15 to 60 min also improved drug solubility. Based on the results from preliminary studies and statistical significance evaluation, carriers such as Pharmacoat, PVP, and PVP/VA, and the milling process through 60 min, were applied in the next step of the studies. The ratio of ETD to carriers was 1:2.

### 3.2. 2nd and 3rd Generation of ETD-SDs

Based on the results from the solubility tests, it was concluded that the use of amorphous polymers provided the highest improvement in ETD solubility. SDs performed with the crystalline compounds (the first generation of SDs) were characterized by a lower ETD solubility and dissolution rate (due to lower Gibbs free energy than amorphous carriers) [7,14,16,22,58]. DSC analysis of the second generation of ETD-SDs detected the possibility of ETD amorphization, in comparison to the first generation, with crystalline carriers. Therefore, Pharmacoat 606, PVP, and PVP/VA were chosen for further studies. Moreover, the Poloxamer was used as a component of SDs to improve their stability and to prevent drug recrystallization. In the next stages of the study, ten SD formulations were prepared: S1–S6 were the third generation of SDs containing ETD, a selected carrier, and a surfactant (Poloxamer), S7–S9 were the second generation of SDs with ETD and a selected carrier, while formulation S10 was a mixture of ETD and Poloxamer. Poloxamer 407 is a block copolymer of ethylene oxide and propylene oxide. It is classified as a non-ionic surfactant, soluble in water and ethanol. It stabilizes and ensures the clarity of liquid drug forms. In addition, as a result of its thermo-surfactant abilities, it has found application in the production of gels and eye drops and as a surfactant compound providing stability in the third generation of SDs [33,58].

#### 3.2.1. Drug Content and Solubility of ETD

The content of ETD in SD formulations (S1–S10) was in the range of 96–101%, i.e., the deviation did not exceed the value of 5%. The results indicated the efficiency of the milling process used for SDs’ preparation and the homogeneity of the obtained powders.

Improvement of ETD’s solubility in water was observed in all formulations, either the second or third generation of SDs, compared to unprocessed ETD (Table 3). Of the second generation of SDs, the highest solubility of ETD in water was shown for Pharmacoat 606 (0.16 mg/mL). Comparing the water solubility of ETD in second (S7–S9) and third generations (S1–S6), a significant increase (*p* < 0.05) was observed in the third generation of SDs. The use of only a surfactant compound (S10) provided a 4-fold increase in the solubility of ETD in water (from 0.065 to 0.262 mg/mL). Literature data [56,59] confirm our results that SDs of the third generation ensured higher solubility and stability than other SD generations. Furthermore, the solubility of ETD in pH 7.4 buffer in the second generation of SDs was similar to that analyzed for the third generation and S10 (Poloxamer; *p* > 0.05).

#### 3.2.2. Dissolution Studies

**Figure 5:**
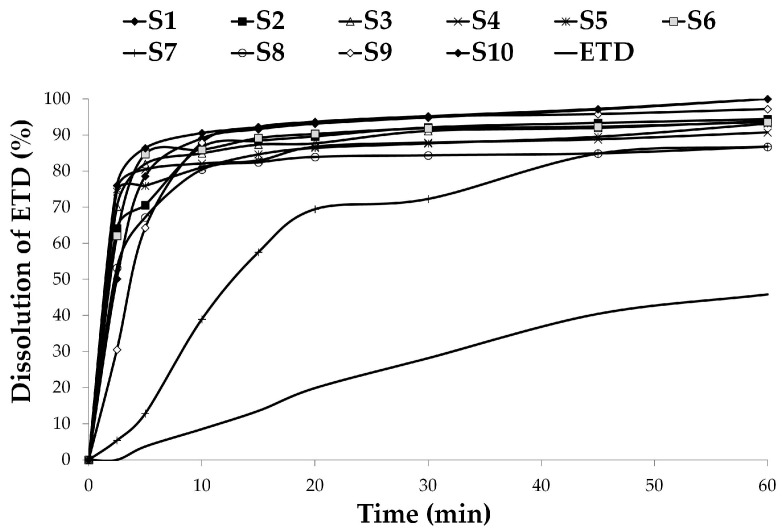
Dissolution profiles of ETD-SDs, compared to unprocessed ETD (control).

In the dissolution study of ETD, the phosphate buffer of pH 7.4 was used as a dissolution medium. It was noticed that an enhanced dissolution rate was observed for all analyzed SDs (Figure 5). The improvement of drug dissolution from SDs was confirmed in many studies [60,61,62,63,64]. Unprocessed ETD exhibited significantly slower and incomplete (45.83%) dissolution, while SDs were characterized by complete or almost complete drug dissolution at the end of the testing time. The maximum and the minimum dissolution rates were recorded for S1, S10 (100%) and S7, S8 (about 86%), respectively, after 60 min of testing. Of all SDs, the dissolution rate was slowest for S7 during 30 min (72.31%), reaching dissolution of 86.81% after 1 h. The improvement in the dissolution rate was noted especially at the beginning of the study (after 2.5 min), when dissolution of more than 70% of ETD from SDs was observed, while no values were recorded for unprocessed ETD.

Formulation S10, containing ETD and Poloxamer, was characterized by the best dissolution profile, and after 2.5 min and 60 min dissolved 76.02% and 100%, respectively. Importantly, S10 was characterized by the lowest particle size. The greatest impact of Poloxamer on the drug solubility and dissolution rate was also reported in the research of Aboutaleb A.E. et al. [65]. On the other hand, the lowest dissolution profile of ETD, at 5.39% (2.5 min) and 86.81% (60 min), was found for S7 (ETD with Pharmacoat). It was shown that SDs containing Poloxamer were characterized by a higher dissolution rate. It was also noted that the concentration of Poloxamer in SDs (33% or 50%) did not affect the dissolution rate. No significant influence on the dissolution rate was observed concerning the type of carrier (*p* > 0.05).

Results of dissolution parameters, such as dissolution efficiency (DE), mean dissolution time (MDT), and similarity factor (f_2_), are presented in Table 4. DE is the ratio of the area under the dissolution–time relationship curve to the area of 100% of the dissolution to the same time point [66]. The mean dissolution time (MDT) characterizes the rate of drug dissolution from the formulation, after the proper time. A higher MDT value suggests a higher potency to retain the drug in the carriers/matrix [67]. The similarity factor (f_2_) compares the dissolution profile of the analyzed formulation to the control (e.g., unprocessed drug). Among the SDs, formulation S10 exhibited the highest DE (0.92%) and low MDT (4.72 min). Unprocessed ETD was characterized by the minimum DE (0.26%) and the maximum MDT (25.51 min). The dissolution rate of ETD-SDs significantly exceeded the results obtained for the unprocessed drug, and the values of the f_2_ were in the range of 8–22. The f_2_ values below 50 indicate significant differences in dissolution profiles between the tested formulations [66]. In this study, SDs containing Poloxamer (S1–S6 and S10) were characterized by better ETD solubility in water than SDs without this component (S7–S9). The f_2_ values of S1–S2 (Pharmacoat with Poloxamer) compared to S7 (Pharmacoat without Poloxamer) and of S5–S6 (PVP/VA with Poloxamer) compared to S9 (PVP/VA without Poloxamer) were below 50. The best dissolution rate was found for formulation S10—ETD with Poloxamer, especially with reference to S7–S9—SD without the addition of Poloxamer (f_2_ < 50). This fact might be due to the properties of the excipient used—Poloxamer 404, which is characterized by surfactant features improving drug solubility and the dissolution rate. Its frequent use and beneficial properties, including SD formulations, has been described in many reports [59,68,69,70].

A kinetic analysis of the dissolution rate was conducted with various mathematical models: zero-order, first-order, and Higuchi models. The value of the correlation coefficient (R^2^) near 1 indicates a fit model to describe the dissolution mechanism. Table 5 presents the R^2^ values for the models and the values of the dissolution rate constant (K). In the study, all SD formulations and unprocessed ETD showed dissolution profiles according to the first-order model (R^2^ from 0.970 to 0.997). Other studies [41,71,72] have shown that the dissolution of poorly soluble drugs from well-soluble systems occurs according to first-order kinetics. Hydrophilic carriers dissolve faster and probably form a boundary layer, from which the drug can dissolve. S10 was characterized by the highest dissolution rate constant (K = 0.509 min^−1^), while unprocessed ETD had the minimum value of K (0.011 min^−1^). Higher K values indicate greater changes in drug concentration with time.

#### 3.2.3. Effect of Carriers and Poloxamer on SD Features—Statistical Analysis

Statistical analysis of the results was conducted using the ANOVA test, and the statistical significance level was set at 95% (α = 0.05). The effects of input factors (carrier type, carrier concentration, and Poloxamer concentration) on ETD solubility in water, solubility in buffer pH 7.4, and the dissolution rate after 60 min were determined. It was found that the concentration of Poloxamer and the carrier had a statistically significant impact (*p* < 0.05) on drug solubility in water, but no significant effect on solubility in buffer and the ETD dissolution profile was noted (*p* > 0.05). All tested SDs (S1–S10) were characterized by similar ETD solubility in phosphate buffer and showed no significant differences. The 3D response surface plots (Figure S4) were created to assess the effect of carrier concentration and Poloxamer on ETD’s solubility in water and buffer pH 7.4, as well as the dissolution rate (measured after 60 min).

#### 3.2.4. Morphological Evaluation

**Figure 6:**
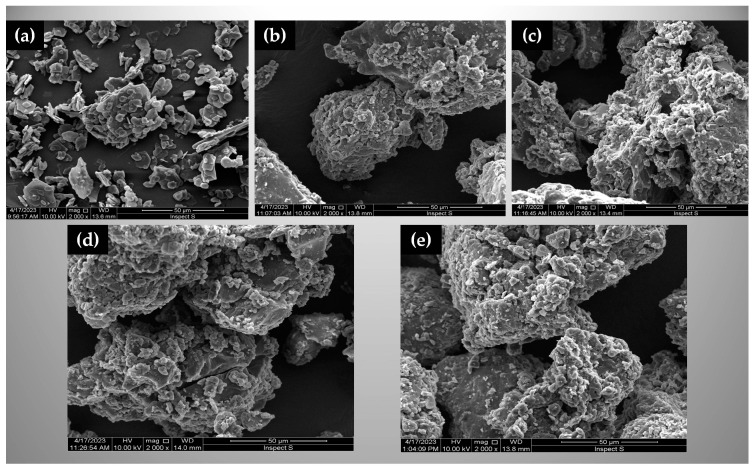
Scanning electron microscopy (SEM) analysis of: (**a**) unprocessed ETD, (**b**) S1 (ETD + Pharmacoat + Poloxamer), (**c**) S3 (ETD + PVP + Poloxamer), (**d**) S5 (ETD + PVP VA + Poloxamer), and (**e**) S10 (ETD + Poloxamer). Magnification 2000×.

Evaluation of SDs by the SEM technique showed a particle size reduction, which can lead to an increase in the drug solubility and dissolution rate [73,74,75]. It can be observed that crystalline ETD particles (Figure 6a) after milling were incorporated and/or dissolved in the carrier matrix. The irregular shape of particles, and their tendency to form aggregates, were also noted (Figure 6).

TEM images (Figure S5) showed that the particle size of SDs prepared with different polymers was in the range of 5.6–114.0 nm. The particle size of S3 and S5 (obtained with PVP/Poloxamer and PVP/VA/Poloxamer, respectively) was the lowest. In TEM pictures, S3 and S5 were observed as spherical particles, S1 was characterized by an irregular shape and the largest particle size, while S10 was different in shape and size structures.

#### 3.2.5. Thermal Analysis

**Figure 7:**
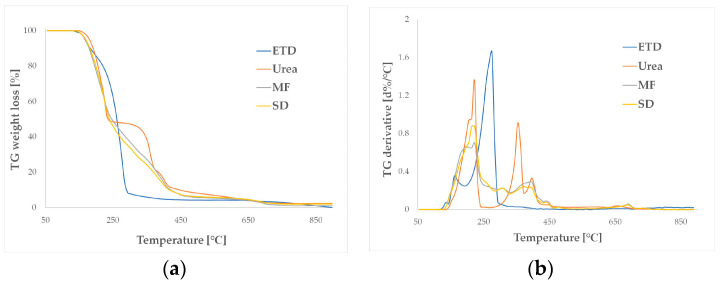
Thermogravimetric (TG) analysis curves of unprocessed ETD, urea, physical mixture (MF) of ETD and urea (1:1), and solid dispersion (SD) of ETD and urea (1:1), presented as: (**a**) TG weight vs. temperature and (**b**) TG derivative vs. temperature.

The thermal properties of formulations containing ETD and urea (physical mixture, SDs) were studied using thermogravimetric analysis (TGA). The drug (ETD) decomposed in one step in the temperature range between 150 and 300 °C. The maximum decomposition rate was at 275 °C. The urea showed three-step decomposition, two significant weight losses of 51% and 28%, at the temperature ranged between 160 and 240 °C, and between 300 and 370 °C, respectively. The last decomposition step started at 370 °C and finished at 700 °C (18% weight loss). In the TGA curves of drug formulations, one decomposition step was observed. The decomposition started at 140 °C and ended at 705 °C, and decomposition of the components was not shown as a separate step. There was no significant difference in the decomposition curve course between the sample prepared as a physical mixture or SDs (Figure 7). The data of TG analysis were similar to the results presented by Zhu N. et al., who studied that thermal degradation of urea occurred in steps: the first at a temperature between 180 and 200 °C, the second stage above 280 °C, and the third stage of decomposition existed at temperatures higher than 380 °C [51].

**Figure 8:**
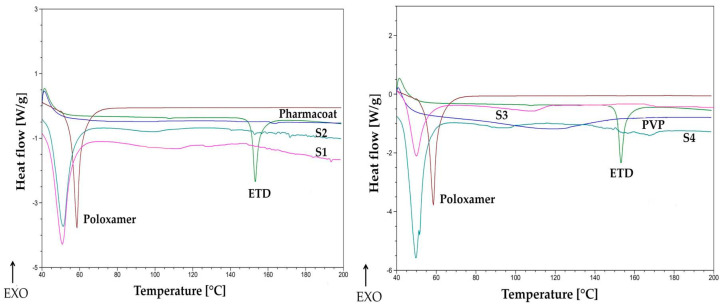

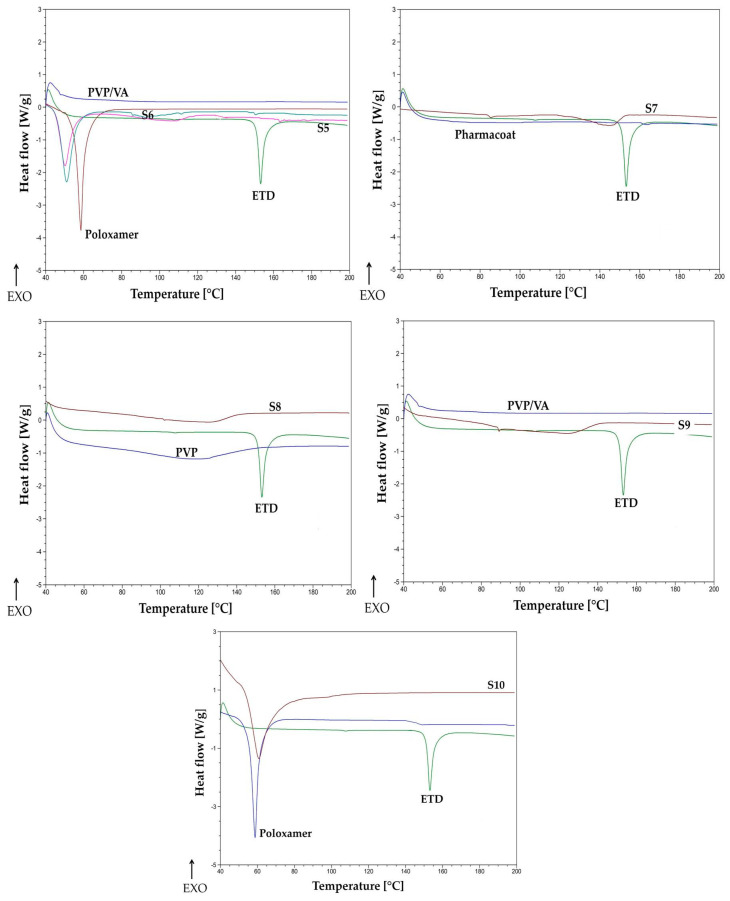
DSC curves of unprocessed drug (ETD), carriers, and SD formulations (S1–S10).

The DSC thermograms of ETD, carriers, and SD (S1–S10) are presented in Figure 8. The pure Poloxamer 407 showed a sharp endothermic peak at about 60 °C, which corresponded to its melting point and is in accordance with the literature data [76,77]. During the DSC analysis, for formulations S1 and S2 (ETD, Pharmacoat 606, and Poloxamer), the loss of the endothermic peak of ETD was observed. This might indicate that the amorphous carrier and surfactant compound, as well as the milling process itself, caused ETD to transition to an amorphous state. Also, no significant difference was observed compared to the DSC curve of the formulation S7, without Poloxamer. Only a slight breaking of the line in the temperature range of 140–150 °C was recorded. The loss and/or reduction of the sharp peak in the DSC image might demonstrate the formation of the amorphous state of ETD, as well as its dissolution in the molten carrier [47,78].

A similar situation was found in the formulations S3–S6, where endothermic peaks were absent, indicating the transition of the drug substance into an amorphous form or dissolution in the carrier. In formulations S8 and S9, where no surfactant was used, the endothermic peak was also reduced. In formulation S10, containing Poloxamer and ETD, the characteristic endothermic peak of unprocessed ETD was not detected.

#### 3.2.6. Attenuated Total Reflectance Fourier Transform Infrared Spectroscopy (ATR-FTIR) Study

FTIR spectra were identified to estimate molecular drug changes as a result of drug–carrier interactions, which could occur in SDs. In the FTIR spectrum of ETD, characteristic signals for the functional groups present in the structure of the drug were found. The narrow, intense signal at 3337 cm^−1^ represented N–H bond stretching vibrations, the bending vibrations of the N-H bond yielded a weak signal at 1617 cm^−1^, and the weak signal at 1144 cm^−1^ could be assigned to C–N stretching vibrations. The C–O bond vibration yielded the narrow signal at 1033 cm^−1^. Vibrations of the carbonyl bond were represented by a strong, narrow signal at 1737 cm^−1^. The signals that originated from the aromatic part of the drug were present at 3055, 1779, 1496, 1033, and 892 cm^−1^, and were interpreted as C–H bond stretching, aromatic combination band, aromatic ring stretching, C–H in-plane bending, and C–H out-of-plane bending, respectively. The C–H aliphatic asymmetric and symmetric bond stretching vibrations were found at 2925 and 2880 cm^−1^, respectively. The C–H bending vibrations were represented by weak, narrow signals at 1496 and 1362 cm^−1^.

In the infrared spectra of carriers, characteristic signals for O–H group stretching (broad signal of hydroxyl group stretching, internally bonded—~3450 cm^−1^), C–O (signal at 1051 cm^−1^ in Pharmacoat 606 for C–O bond present in the piranosyl ring) and C=O bond stretching (~1650 cm^−1^ for amide bond and 1730 cm^−1^ for ester bond), C–H symmetric and asymmetric stretching (~2940 cm^−1^ and ~2880 cm^−1^, respectively), and N–H stretching and N-H bending (characteristic for urea at 3425 cm^−1^ and 1591 cm^−1^) were found.

**Figure 9:**
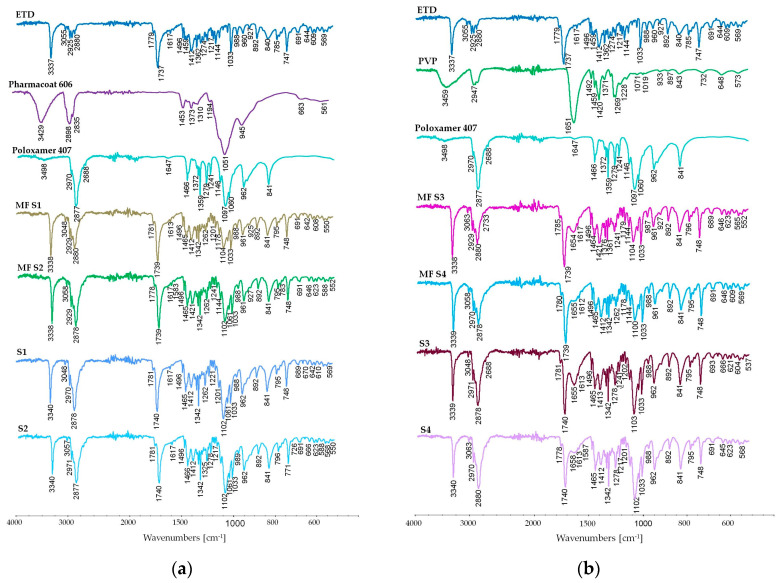

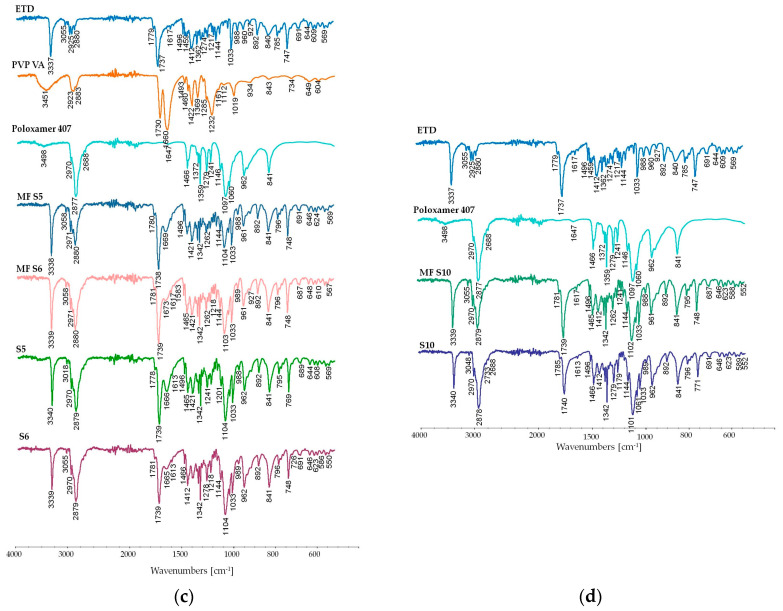
FTIR spectra of unprocessed drug (ETD), used carriers, physical mixtures (MF), and SDs. The spectra refer to SDs: (**a**) S1 and S2, (**b**) S3 and S4, (**c**) S5 and S6, and (**d**) S10.

In the spectra of the physical mixtures as well as SDs of ETD, signals characteristic of the drug were observed, which indicated compatibility of the drug with the tested carriers. In all spectra, extinction of a broad signal of O–H bond stretching was observed. This signal represented intramolecular interactions between hydroxyl groups present in the carriers, and the shortage of this signal indicated changes in the organization of compounds present in the mixtures and dispersions. The spectra of FTIR are presented in Figure 9 and Figure S6.

**Figure 10:**
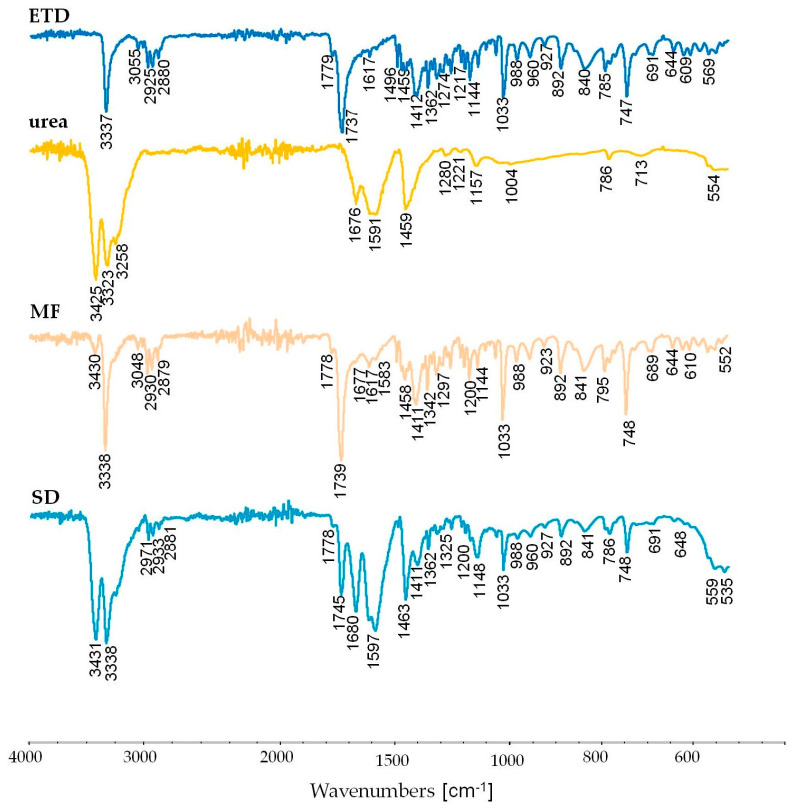
FTIR spectra of unprocessed drug (ETD), urea, physical mixtures (MF), and solid dispersion (SD).

Due to the fact that during thermal analysis of SDs with urea, the changes in the DSC curve and urea degradation in the TG evaluation were noted, FTIR studies were additionally conducted (Figure 10). Some differences in the spectra of SD and MF (physical mixture) of ETD and urea were observed. It was noted that spectra of SD signals at 3431, 1597, and 1463 cm^−1^ were stronger than in the physical mixture, and a strong signal at 1680 cm^−1^ appeared—a shifted signal from urea. The intensification and the shift of the latter signal might result from the hydrogen bond formation between ETD and urea [79].

## 4. Conclusions

ETD-SDs with various carriers were prepared via the ball-milling technique to overcome the problems associated with the poor solubility of ETD. All designed SDs showed better ETD water solubility and a faster dissolution rate compared to unprocessed drugs. SDs prepared with amorphous carriers (HPMC, PVP, and PVP/VA) were characterized by higher solubility than dispersions with crystalline features (urea and mannitol). The addition of Poloxamer to the SD matrix ensured a greater ETD solubility and dissolution rate. FTIR spectra confirmed the compatibility of ETD with the carriers (PVP, hypromellose, PVP/VA, and Poloxamer). The results indicated that the optimal SDs for ETD should contain Poloxamer as the alone component (such as S10) or as the additive (S1–S6). Identification of the physicochemical properties of ETD-SDs and statistical analysis of the results provided essential information about the optimal composition of SDs for ETD.

## Figures and Tables

**Table 1:** Composition of the designed SDs with ETD (ETD-SDs).

ETD-SD	Ratio of ETD:Carrier	Time of Milling (min)
F1, F2, F3	1:0	15, 30, 60
PVP		
F4, F5, F6	2:1	15, 30, 60
F7, F8, F9	1:1	15, 30, 60
F10, F11, F12	1:2	15, 30, 60
Pharmacoat		
F13, F14, F15	2:1	15, 30, 60
F16, F17, F18	1:1	15, 30, 60
F19, F20, F21	1:2	15, 30, 60
Urea		
F22, F23, F24	2:1	15, 30, 60
F25, F26, F27	1:1	15, 30, 60
F28, F29, F30	1:2	15, 30, 60
Mannitol		
F31, F32, F33	2:1	15, 30, 60
F34, F35, F36	1:1	15, 30, 60
F37, F38, F39	1:2	15, 30, 60
PVP/VA		
F40, F41, F42	2:1	15, 30, 60
F43, F44, F45	1:1	15, 30, 60
F46, F47, F48	1:2	15, 30, 60

**Table 2:** Composition of SDs with ETD (S1–S10).

ETD-SD	ETD (g)	Polymer (g)	Poloxamer (g)
Pharmacoat			
S1	1	1	1
S2	1	0.5	1.5
PVP			
S3	1	1	1
S4	1	0.5	1.5
PVP/VA			
S5	1	1	1
S6	1	0.5	1.5
Pharmacoat			
S7	1	2	0
PVP			
S8	1	2	0
PVP/VA			
S9	1	2	0
Poloxamer 407			
S10	1	0	2

**Table 3:** ETD solubility in water and in phosphate buffer pH 7.4.

Formulation	Solubility in Water (mg/mL) *	Solubility in Phosphate Buffer pH 7.4 (mg/mL) **
S1 (Pharmacoat:Poloxamer = 1:1)	0.19 ^a^ ± 0.01	1.69 ± 0.02
S2 (Pharmacoat:Poloxamer = 0.5:1.5)	0.25 ^b^ ± 0.00	1.77 ± 0.03
S3 (PVP:Poloxamer = 1:1)	0.19 ^ca^ ± 0.01	1.66 ± 0.06
S4 (PVP:Poloxamer = 0.5:1.5)	0.16 ^dc^ ± 0.01	1.64 ± 0.13
S5 (PVP/VA:Poloxamer = 1:1)	0.21 ^eac^ ± 0.01	1.77 ± 0.07
S6 (PVP/VA:Poloxamer = 0.5:1.5)	0.22 ^fbe^ ± 0.01	1.86 ± 0.04
S7 (Pharmacoat)	0.16 ^gd^ ± 0.01	1.73 ± 0.07
S8 (PVP)	0.12 ^h^ ± 0.01	1.68 ± 0.03
S9 (PVP/VA)	0.14 ^idgh^ ± 0.01	1.73 ± 0.02
S10 (Poloxamer)	0.26 ^jb^ ± 0.01	1.82 ± 0.11

* Different letters (a–j) in the column mean significant differences (*p* < 0.05). All formulations (S1–S10) with regard to ETD solubility (0.06 mg/mL) showed statistical significance at the *p* < 0.001 level. ** Statistical significance (*p* < 0.05) was shown for S3 with respect to S6, S4 to S6, and S2, S5, S6, S7, S9, and S10 in relation to the solubility of unprocessed ETD in buffer (1.51 mg/mL).

**Table 4:** Measured responses (DE and MDT) and similarity factors (f_2_) of SDs (S1–S10) and unprocessed ETD.

Formulation	Dissolution Parameters		Similarity Factor, f_2_
	DE (%)	MDT (min)	
S1	0.90	5.87	9.7
S2	0.87	4.58	10.4
S3	0.87	4.35	9.9
S4	0.85	4.04	10.5
S5	0.85	5.42	10.6
S6	0.88	3.80	9.9
S7	0.65	15.15	22.8
S8	0.80	4.57	12.8
S9	0.88	5.72	10.9
S10	0.92	4.72	8.4
ETD	0.26	25.51	-

**Table 5:** Kinetic analysis of data obtained in the dissolution test for ETD-SDs and unprocessed ETD.

Formulation	Correlation Coefficient (R^2^)			K * (min^−1^)
	Zero Order	First Order	Higuchi Model	
S1	0.632	0.995	0.821	0.276
S2	0.599	0.984	0.795	0.296
S3	0.545	0.986	0.744	0.404
S4	0.514	0.976	0.713	0.437
S5	0.553	0.970	0.745	0.393
S6	0.549	0.991	0.752	0.360
S7	0.885	0.985	0.958	0.048
S8	0.596	0.975	0.796	0.209
S9	0.668	0.995	0.847	0.184
S10	0.542	0.993	0.739	0.509
ETD	0.989	0.997	0.969	0.011

* K—dissolution rate constant.

## Data Availability

The data supporting the reported results are available upon request from the corresponding author. The collected data can be found in the archived datasets, at the following link: https://doi.org/10.18150/EBVQ4R.

## Supplementary Materials

The following supporting information can be downloaded at: https://www.mdpi.com/article/10.3390/ma17163923/s1. Figure S1: DSC curves of pure ETD without carriers, after milling for 15 min (F1), 30 min (F2), and 60 min (F3). Figure S2: DSC curves of ETD-SDs with Pharmacoat 606 and ETD as a control. The drug:carrier ratios were 2:1 (F13–F15), 1:1 (F16–F18), and 1:2 (F19–F21). Figure S3: DSC curves of ETD-SDs with PVP/VA and ETD as a control. The drug:carrier ratios were 2:1 (F40–F42), 1:1 (F43–F45), and 1:2 (F46–F48). Figure S4: 3D response surface plots: effect of carrier and Poloxamer concentration on (a) ETD solubility in water, (b) ETD solubility in buffer pH 7.4, and (c) dissolution rate (measured after 60 min). Figure S5: TEM micrographs of SD: (a) S1 (ETD + Pharmacoat + Poloxamer), (b) S3 (ETD + PVP + Poloxamer), (c) S5 (ETD + PVP VA + Poloxamer), and (d) S10 (ETD + Poloxamer). Figure S6: FTIR spectra of unprocessed drug (ETD), used carriers, physical mixtures (MF), and SDs. The spectra refer to SDs: (a) S7, (b) S8, and (c) S9.
