# Supplementary material for: Solid Dispersions Obtained by Ball Milling as Delivery Platform of Etodolac, a Model Poorly Soluble Drug
Source: Materials (Basel). 2024 Aug 7;17(16):3923. doi: 10.3390/ma17163923 (PMC11355714; doi:10.3390/ma17163923)
Supplement: Supplementary file 1 [file materials-17-03923-s001.zip › materials-3120668-supplementary.pdf]

Supplementary Materials

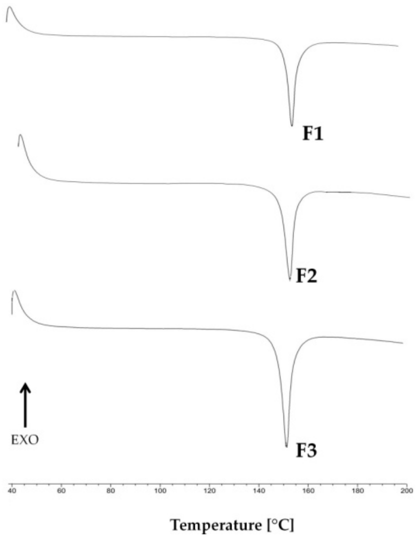

Figure S1. DSC curves of pure ETD without carriers, after milling for 15 min (F1), 30 min (F2) and 60 min (F3).

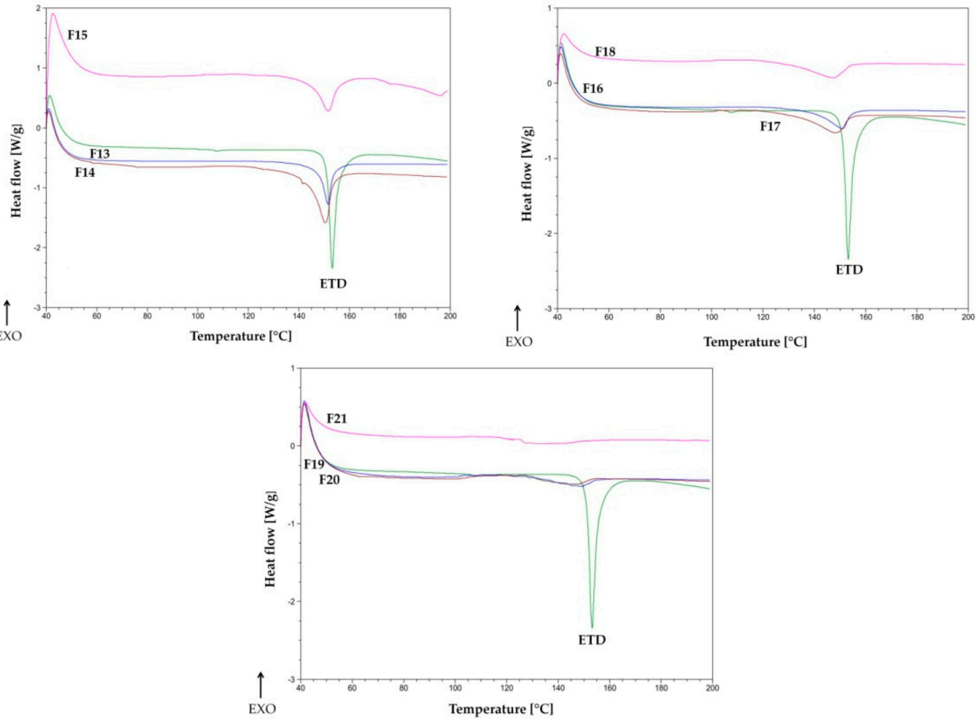

Figure S2. DSC curves of ETD-SD with Pharmacoat 606 and ETD as control. The drug:carrier ratios are 2:1 (F13 - F15), 1:1 (F16 - F18) and 1:2 (F19 - F21).

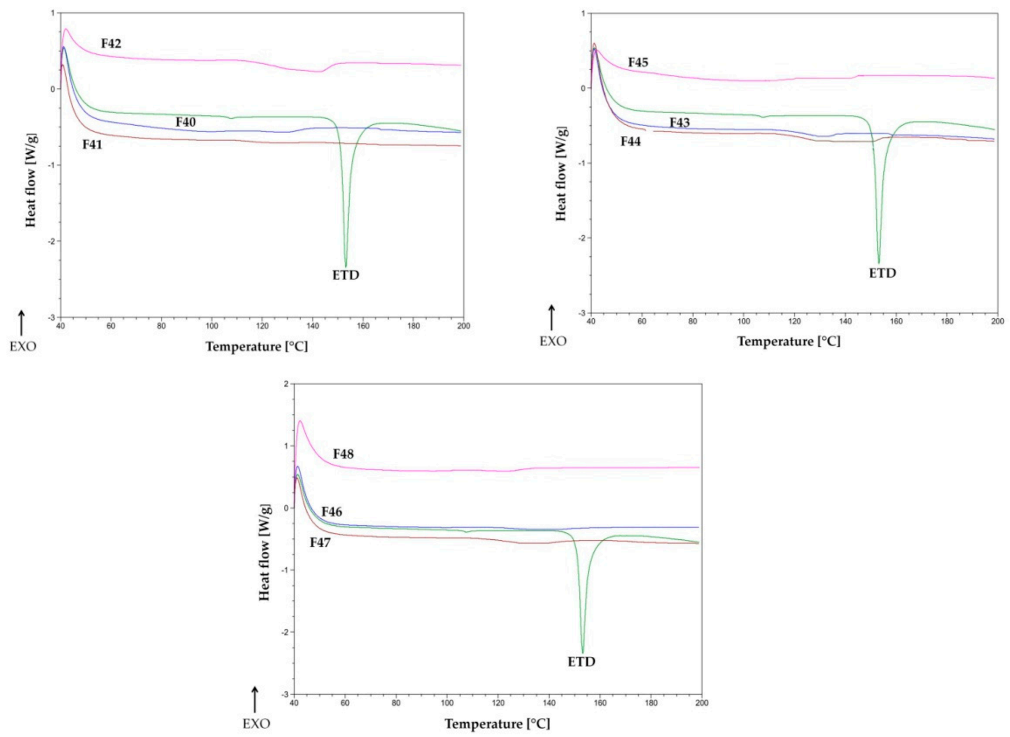

Figure S3. DSC curves of ETD-SD with PVP/VA and ETD as control. The drug:carrier ratios are 2:1 (F40 - F42), 1:1 (F43 - F45) and 1:2 (F46 - F48).

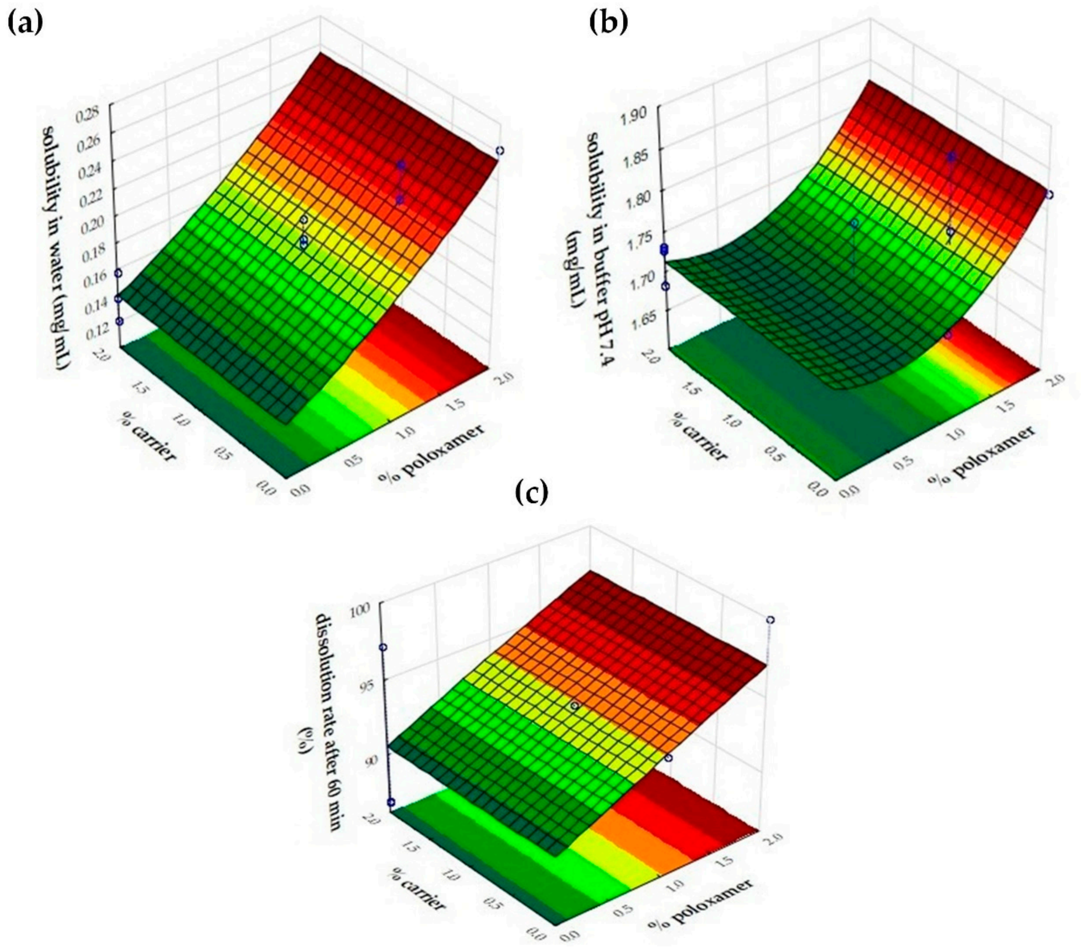

Figure S4. 3D response surface plots, effect of carrier and poloxamer concentration on: (a) ETD solubility in water; (b) ETD solubility in buffer pH 7.4; (c) dissolution rate (measured after 60 min).

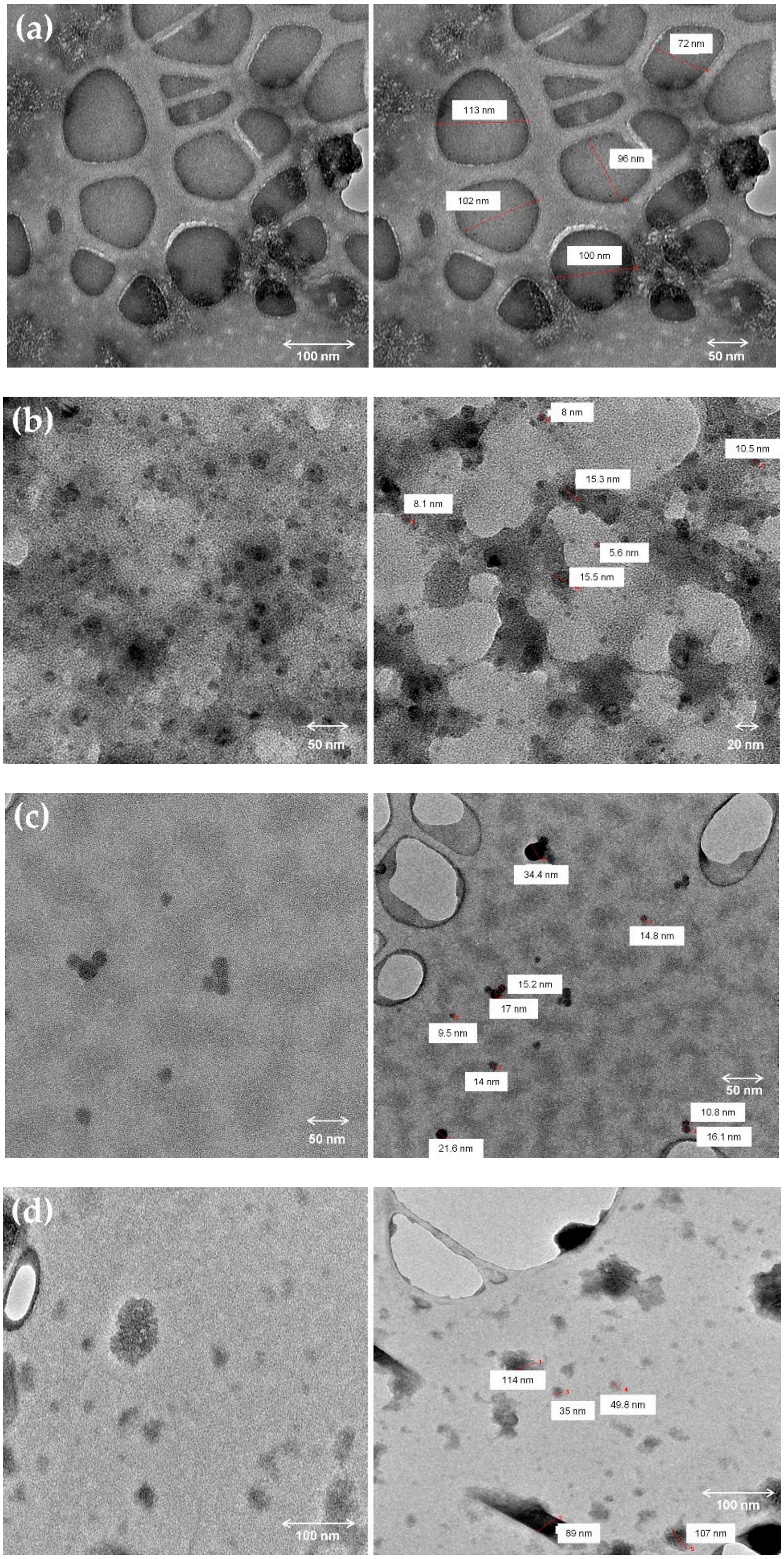

Figure S5. TEM micrographs of SD: (a) S1 (ETD + Pharmacoat + poloxamer); (b) S3 (ETD + PVP + poloxamer); (c) S5 (ETD + PVP VA + poloxamer); (d) S10 (ETD + poloxamer).

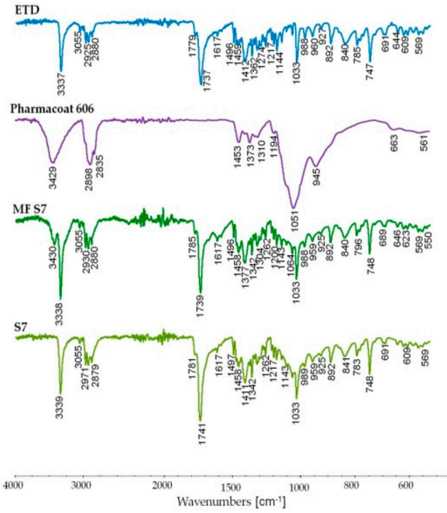

(a)

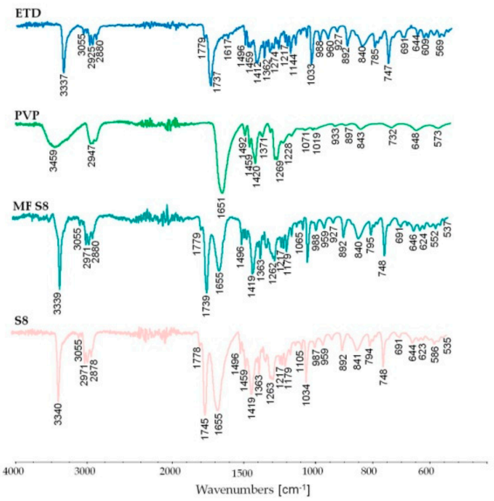

(b)

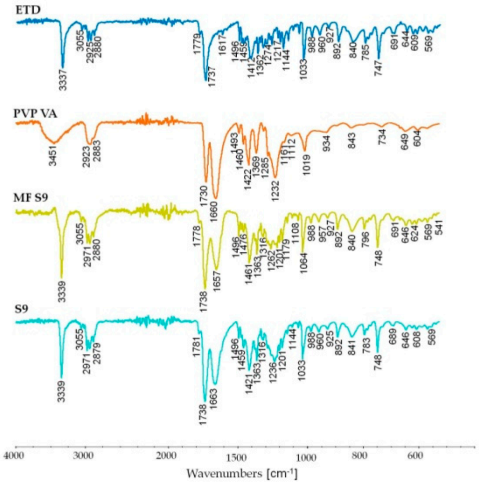

(c)

Figure S6. FTIR spectra of unprocessed drug (ETD), used carriers, physical mixtures (MF) and SD. The spectra refer to SD: **(a)** S7; **(b)** S8; **(c)** S9.
